# Pluronic based β-cyclodextrin polyrotaxanes for treatment of Niemann-Pick Type C disease

**DOI:** 10.1038/srep46737

**Published:** 2017-04-28

**Authors:** Christopher J. Collins, Bradley P. Loren, Md Suhail Alam, Yawo Mondjinou, Joseph L. Skulsky, Cheyenne R. Chaplain, Kasturi Haldar, David H. Thompson

**Affiliations:** 1 Department of Chemistry, Purdue University, Multi-disciplinary Cancer Research Facility, 1203 W, State Street, West Lafayette, Indiana 47907, United States; 2 Boiler-Parseghian Center for Rare and Neglected Diseases, University of Notre Dame, Notre Dame, IN 46556, USA; 3 Department of Biological Sciences, University of Notre Dame, 103 Galvin Life Sciences, Notre Dame, IN 46556, USA; 4 Purdue University Center for Cancer Research, 201 S, University Street, West Lafayette, Indiana 47907, United States; 5 Weldon School of Biomedical Engineering, Purdue University, 206 S, Martin Jischke Drive, West Lafayette, Indiana 47907, United States

## Abstract

Niemann-Pick Type C disease (NPC) is a rare metabolic disorder characterized by disruption of normal cholesterol trafficking within the cells of the body. There are no FDA approved treatments available for NPC patients. Recently, the cycloheptaglucoside 2-hydroxypropyl-β-cyclodextrin (HP-β-CD) has shown efficacy as a potential NPC therapeutic by extending lifetime in NPC mice, delaying neurodegeneration, and decreasing visceral and neurological cholesterol burden. Although promising, systemic HP-β-CD treatment is limited by a pharmacokinetic profile characterized by rapid loss through renal filtration. To address these shortcomings, we sought to design a family of HP-β-CD pro-drug delivery vehicles, known as polyrotaxanes (PR), capable of increasing the efficacy of a given injected dose by improving both pharmacokinetic profile and bioavailability of the HP-β-CD agent. PR can effectively diminish the cholesterol pool within the liver, spleen, and kidney at molar concentrations 10-to-100-fold lower than monomeric HP-β-CD. In addition to this proof-of-concept, use of PR scaffolds with differing physiochemical properties reveal structure-activity relationships in which PR characteristics, including hydrophobicity, threading efficiency and surface charge, were found to both decisively and subtly effect therapeutic efficacy. PR scaffolds exhibit absorption, pharmacokinetics, and biodistribution patterns that are significantly altered from monomeric HP-β-CD. In all, PR scaffolds hold great promise as potential treatments for visceral disease in NPC patients.

Niemann-Pick Type C disease (NPC) is a panethnic, heterogeneous, and extremely rare metabolic disorder (estimated incidence of approximately 1:120,000 live births^1^) characterized by a disruption of normal cholesterol trafficking within cells^2^. Natural transport is hindered by mutations in either the NPC1 or NPC2 proteins in the late endosomal/lysosomal (LE/LY) system. Genetic mutation in the NPC1 gene is implicated in ~95% of clinical cases^2^,^3^. These proteins are responsible for the movement of unesterified cholesterol (UC) through the LE/LY, with their disruption resulting in aberrant accumulation of UC and other lipids including glycolipids and fatty acids within these compartments^4^,^5^,^6^. This defect presents a range of visceral and neurological symptoms including organomegally of the spleen and liver, supranuclear gaze palsy, delayed motor development, seizures, and dementia^7^,^8^. The progression of these NPC symptoms are ultimately fatal.

There are no United States Food and Drug Administration (FDA) approved treatments currently available for NPC patients. In 2009, miglustat (Zavesca), an iminosugar small molecule drug that is currently approved to treat Gaucher’s disease, was approved by the European Agency of Medicines for use in NPC, despite being rejected by the FDA^9^. The treatment has been shown to slow neurodegeneration in NPC mice and NPC patients, despite ultimately being unable to alter disease progression^10^,^11^. Small molecule drugs known as histone deacetylase inhibitors (HDACi) have also been shown to reduce UC accumulation in NPC cells, including vorinostat, an HDACi that has been previously approved by the FDA for the treatment of cutaneous T-cell lymphoma^12^,^13^,^14^. 2-Hydroxypropyl-β-cyclodextrin (HP-β-CD) and other β-cyclodextrin (CD) derivatives have also shown efficacy toward UC mobilization in cell and animal models of NPC^15^,^16^,^17^,^18^.

Upon *in vivo* administration in mouse and cat models of NPC, CD derivatives have a number of beneficial effects. A single intraperitoneal (IP) injection of HP-β-CD early in life leads to an increase in average overall lifetime of NPC mice by as much as 50%^15^. In addition, neurodegeneration is significantly delayed^15^. A number of studies have shown the ability of HP-β-CD to decrease total UC burden in many visceral organs, including liver, spleen and kidney^16^,^17^. Serial HP-β-CD IP injection is even more beneficial, although no effect is seen in lung tissue in any case, nor in the brain tissue of older mice without intrathecal injection^15^,^17^,^18^,^19^,^20^. Similar beneficial response to HP-β-CD treatment has been reported in the NPC cat model^21^. Although promising, HP-β-CD treatment for NPC has significant shortcomings. These are largely a result of poor CD pharmacokinetics and bioavailability, particularly in brain since HP-β-CD does not effectively cross the blood-brain barrier. Large percentages of the injected dose are rapidly lost through renal filtration and found unmetabolized in the urine^19^,^22^. In 49-day old mice, 90% of the dose is cleared from the body within 6 h after subcutaneous injection^19^,^23^. This pharmacokinetic profile will limit the effectiveness of HP-β-CD by systemic injection, necessitating the administration of increased drug concentrations at more frequent intervals. Ototoxicity in NPC models has also been reported^18^. Currently, translation of HP-β-CD therapy for treatment of brain in NPC patients via intrathecal administration is underway to obviate these limitations, with the drug formulation currently undergoing a Phase 2b/3 trial (https://clinicaltrials.gov/ct2/show/NCT02534844).

To address the shortcomings of HP-β-CD treatment, we sought to design a high molecular weight, pro-drug form of CD, known as polyrotaxanes (PR), that should be capable of increasing the efficacy of a given injected dose and obviate non-specific UC extraction by blocking the CD cavity until PR activation in the LE/LY. PR are comprised of macrocycles, in this case CD derivatives, that are non-covalently threaded onto polymeric cores before the addition of terminal blocking substituents to prevent CD loss from the central polymer (Fig. 1a). PR can be synthesized utilizing a variety of polymer cores and macrocycles, yielding materials with vastly different physiochemical properties. A PR carrying negatively charged 4-sulfobutylether-β-CD (SBE-β-CD), for example, shows greatly enhanced water solubility^24^. These vehicles should attain a large hydrodynamic radius to prevent rapid renal excretion, increase CD residence time within the body, and improve the overall pharmacokinetics of each injected dose to serve as a long circulating CD delivery system.

We have previously shown in mice that PR are able to circulate for extended periods *in vivo* after intravenous (IV) injection in comparison to monomeric HP-β-CD^25^,^26^. Additionally, in cell culture models of NPC, PR materials effectively mobilize UC from sites of abnormal sequestration^24^,^27^,^28^,^29^. This has been achieved using a variety of Pluronic triblock co-polymer cores (i.e., poly(ethylene glycol) (PEG) blocks separated by a poly(propylene glycol) (PPG) core) and CD derivatives, including HP-β-CD, unmodified β-CD, and negatively charged SBE-β-CD^24^. *In vitro,* PR materials are more effective toward UC mobilization, on a molar basis, than monomeric HP-β-CD. In this study, we sought to evaluate our CD PR derivatives in the mouse model of NPC disease. We utilized a family of PR materials, consisting of differing polymer cores and β-CD macrocycles, in an effort to define relationships between material physiochemical properties and the ability of the respective PR to reduce the total UC burden in the organs of NPC1^nih^ mice (Fig. 1). Therapeutic efficacy was then compared to benchmark concentrations of monomeric HP-β-CD. In addition, because it has been shown that nanomaterials can affect cytochrome P450 (CYP)-mediated metabolism, and since NPC patients have inherently diminished CYP activity^30^,^31^, we investigated possible toxic effects of selected PR scaffolds on CYP isozymes. Included in this analysis were the prevalent CYP isoforms 3A4, 2D6, and 2C9, collectively responsible for ~90–95% of all drug metabolism^30^. Finally, we investigated the kinetics of PR absorption from the IP space and the resulting pharmacokinetics and biodistribution after IP administration. To our knowledge, this is the first report of PR-mediated NPC therapy *in vivo*.

## Results

### PR Characterization

PR derivatives under evaluation were selected to exhibit a range of physiochemical properties (Table 1). Two of the PR featured cores comprised of the high-molecular weight, relatively hydrophilic Pluronic F127. Due to its extensive PEG blocks, 200 units total, this copolymer has a hydrophilic-lipophilic balance (HLB) of 22. In contrast, the remaining two PR utilized a hydrophobic and low molecular weight core, Pluronic L81. This copolymer features an extensive central PPG block, 43 units on average, relative to its small PEG blocks of 6.25 units (HLB = 2). Within these two groups, one derivative carried only neutral HP-β-CD, while the other incorporated a mixture of HP-β-CD and negatively charged SBE-β-CD. Consequently, the overall PR characteristics differed greatly depending on the starting materials utilized. Total PR molecular weights ranged from 24 kDa–47 kDa. L81 PR characteristics varied extensively, with 24 kDa and 60.5% threading for L81-HP PR, compared to 47 kDa and ~100% threading for the mixed L81-HP/SBE PR. In contrast, the F127 series was consistent in its MW and threading efficiency. The F127-HP and F127-HP/SBE PR were 31 kDa and 32 kDa, respectively, with threading efficiencies of 33.8% and 30.8%.

In general, threading efficiency was dependent on polymer core characteristics, with more hydrophilic polymers threading less effectively. The high threading of L81-HP/SBE may be explained by selective dialysis of minimally threaded PR during purification against 6–8 kDa molecular weight cutoff (MWCO) membranes. Consequently, PR materials bearing ≤3 CD macrocycles would still be susceptible to loss by migration across the membrane. Further, increased charge density on the PR surface due to SBE incorporation would decrease or prevent PR association in solution, thereby preventing PR retention through micellization. Finally, percent threading calculations are based on the reported average molecular weight of the PPG core, meaning that cores of higher molecular weight would have the ability to carry a greater number of CDs than suggested by the global polymer MW average. These materials would be more likely to be retained during purification and contribute to an increase in apparent threading efficiency. The property variation inherent in this family of materials enabled the study of multiple effects on PR therapeutic efficacy including polymer molecular weight and hydrophobicity, PR threading efficiency, and β-CD derivative charge.

### LC/MS/MS Method Linearity and Precision

Analysis of organ cholesterol pools was performed using LC/MS/MS and d_7_-cholesterol as an internal standard. Once extracted from sample tissue, cholesterol was derivatized with picolinic acid according to Honda *et al*.^32^ Chromatographic separation was performed using a Vydac C4 column. Linearity of the tissue cholesterol analysis was confirmed by monitoring cholesterol concentrations with progressively increasing injection volumes. Data analysis was performed by linear regression and was found to be linear over the concentration range studied (Supplementary Table S1). Inter-assay precision was studied by comparison of extracted UC aliquots subjected to derivatization and analysis on separate days. Final cholesterol concentrations obtained were very consistent and differed by <6% in every case (Table 2). Representative chromatograms can be found in Supplementary Figures S1 and S2.

### Cholesterol Reduction

PR were administered to NPC^nih^ mice by IP injection at an effective concentration of 400 mg/kg CD derivative. In addition to the PR, treatment groups included an untreated control and monomeric HP-β-CD at 400 mg/kg and 4,000 mg/kg for comparison with previously reported treatments. Treatments with HP-β-CD monomer at these concentrations have shown varying degrees of therapeutic efficacy, in reducing cholesterol accumulation and extending lifetime, and would be taken as appropriate benchmarks for PR performance^15^,^18^,^33^. This approach meant that the PR materials were administered at lower molar concentrations overall in every case.

**Figure 2:**
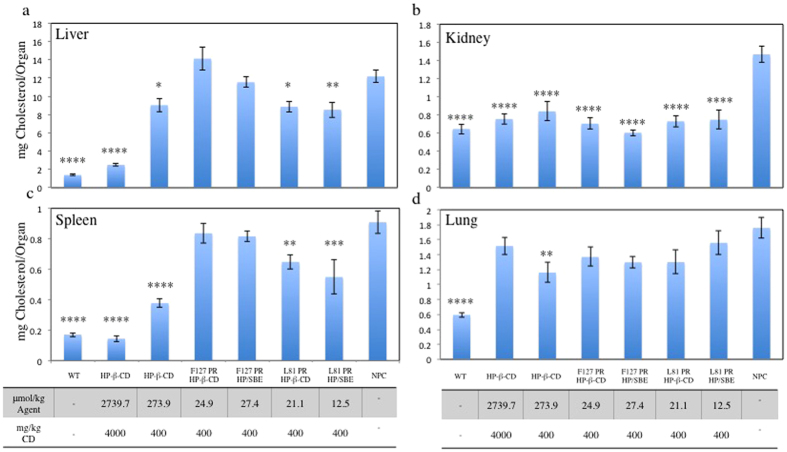
PR are capable of reducing cholesterol accumulation in the visceral organs of NPC^nih^ mice. Unesterified cholesterol extracted from the visceral organs was quantified by LC/MS/MS. Cholesterol concentrations in the (**a**) liver, (**b**) kidney, (**c**) spleen, and (**d**) lung are shown. For WT control, n = 7. For all others, n = 6. Statistical analyses are relative to untreated NPC control. *p = 0.05, **p = 0.01, ***p = 0.005, ****p = 0.001.

Significant differences in PR therapeutic efficacy are evident in the liver. Here, only the L81-based PR are capable of reducing UC accumulation. The reductions were equivalent to 400 mg/kg (273.9 μmol/kg) monomeric HP-β-CD, despite administration of L81-HP PR and L81-HP/SBE PR at 13-fold and 22-fold lower molar concentrations (Fig. 2a), respectively. These materials reduced UC accumulation to 8.9 and 8.5 mg/organ, respectively, from an untreated NPC^nih^ level of 12.2 mg/organ. In contrast, F127-HP PR and F127-HP/SBE PR were ineffective at mobilizing stored UC in liver.

This pattern was identical for spleen UC levels, with L81PR exhibiting enhanced therapeutic efficacy relative to F127PR. Both L81-HP PR and L81-HP/SBE PR significantly reduced UC accumulation, with the charged SBE-carrying PR being slightly more effective, on average, relative to untreated NPC^nih^ controls (Fig. 2c). In this case, a 400 mg/kg (273.9 μmol/kg) dose of monomeric HP-β-CD was more effective than all PR, with 4000 mg/kg (2739.7 μmol/kg) monomeric HP-β-CD returning spleen UC concentrations to normal levels. This discrepancy may reflect differences in biodistribution, with highly threaded and hydrophobic L81PR preferentially depositing in the liver and exhibiting increased hepatic clearance.

In the kidney, all PR scaffolds were equally effective toward total UC reduction (Fig. 2b). PR treatment brought renal UC to levels similar to WT controls treated with 4,000 mg/kg (2739.7 μmol/kg) monomeric HP-β-CD, despite being administered at a ≥100-fold lower molar concentration. There are no apparent differences in PR efficacy in this tissue regardless of the overall PR physiochemical properties. This may indicate a common clearance pathway amongst the materials, with renal filtration playing a significant role regardless of charge, molecular weight, or hydrophobicity.

UC pools in the lung (Fig. 2d) were unaffected by administration of either monomeric HP-β-CD or any of the PR species. UC concentrations in NPC^nih^ animals, whether treated or untreated, ranged from 1.2–1.6 mg/organ. WT mice exhibited pulmonary UC concentrations of ~0.6 mg/organ, consistent with previous reports showing pulmonary sterol pools to be unresponsive to monomeric HP-β-CD treatment^23^,^34^.

**Figure 3:**
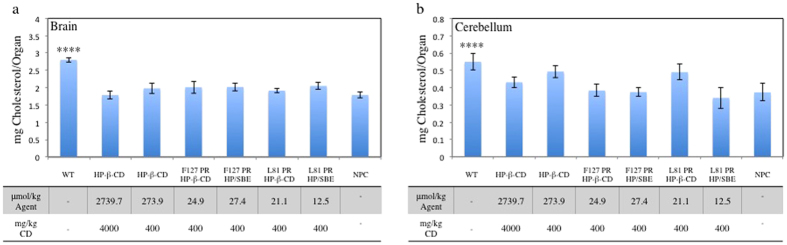
Cholesterol concentrations in the central nervous system of NPC^nih^ mice are unaffected by PR treatment. Unesterified cholesterol concentrations in the (**a**) brain and the (**b**) cerebellum specifically were determined by LC/MS/MS after extraction. For WT control n = 7. For all others n = 6. Statistical analyses are relative to untreated NPC control. ****p = 0.001.

Similarly, UC content in the brain or cerebellum of NPC^nih^ mice was not affected by IP treatment with either PR or monomeric HP-β-CD (Fig. 3a and b). UC concentrations for these organs were ~1.8–2 mg/organ and ~0.43–0.49 mg/organ, respectively, with no statistical difference between the NPC^nih^ control and treatment groups. These levels are slightly lower than the unaffected WT mouse, where the UC concentrations were 2.8 and 0.5 mg/organ, respectively. This effect is consistent with previous reports and has been attributed to neuronal death with increasing age and disease severity^15^,^34^. Over time this reduction in cell number leads to a decrease in total brain cholesterol, while the remaining cells will continue to have increased lysosomal concentrations individually. Lack of therapeutic efficacy is likely due to the inability of either monomeric HP-β-CD or PR to permeate the blood brain barrier. The lack of CD access to the CNS has driven the need for intrathecal injections of monomeric HP-β-CD in mouse models and in the clinic to prevent neurodegeneration^19^,^21^. Unfortunately, this transport deficiency is not improved by rotaxanation of HP-β-CD in the PR forms reported here.

### PR Effects on Cytochrome P450 Metabolism

**Figure 4:**
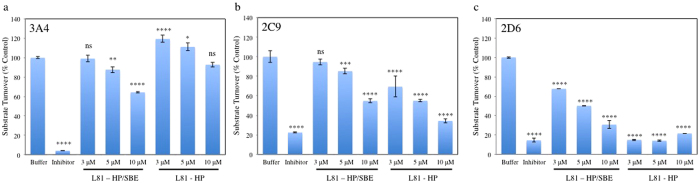
Incubation with L81-HP PR and L81-HP/SBE PR affect the substrate turnover of Cytochrome P450 isoforms. Isoforms studied included (**a**) 3A4, (**b**) 2C9, and (**c**) 2D6. Studies characterized the cleavage of fluorogenic CYP substrates in the presence of PR compounds or known inhibitors ketoconazole (3A4), sulphaphenazole (2C9), and quinidine (2D6). The intensity of fluorescence indicates the extent of CYP activity perturbation. Experiments were done in triplicate and expressed as a percent of uninhibited control. *p = 0.05, **p = 0.01, ***p = 0.005, ****p = 0.001.

Given the PR biodistribution and the fact that L81PR were able to reduce visceral cholesterol levels in the spleen and liver, we assessed the effects that PR administration may have on CYP function. Since CYPs are critical elements of drug metabolism, we focused our attention on the most important of these enzymes, CYP 3A4, 2D6, and 2C9. PR administration had the least impact on CYP 3A4 function (Fig. 4). Low concentrations of HP/SBE PR had no effect on substrate turnover. At concentrations higher than 5 μM, however, a concentration dependent decrease in 3A4 activity was observed. Low concentrations of L81-HP PR appear to have a stimulatory effect on 3A4 turnover, with 3 μM and 5 μM PR incubations resulting in enhanced substrate turnover. Conversely, CYP 2C9 is more acutely affected by PR administration. L81-HP/SBE PR elicited a concentration dependent inhibition that was identical to 3A4 at 3 μM and 5 μM concentrations. At 10 μM L81-HP/SBE PR, however, this caused increased inhibition with only 55% substrate turnover. L81-HP PR inhibited 2C9 at all tested concentrations, with turnover ranging from 69% for 3 μM PR to 34% for 10 μM PR. Finally, there was significant inhibition of CYP 2D6 for both PR at all concentrations. L81-HP/SBE PR inhibition followed a concentration dependent drop from 68% to 31% turnover, while L81-HP limited 2D6 to just 14–21% turnover. These effects are different from, and more substantial than, those exhibited by their precursor starting materials, HP-β-CD, SBE-β-CD, and Pluronic L81 (Supplementary Figure S3).

### Magnetic Resonance Imaging

**Figure 5:**
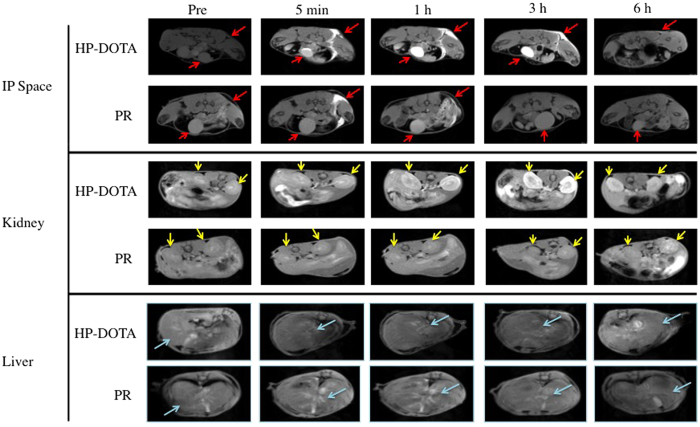
Magnetic resonance images of Balb/c mice after IP administration of Gd^3+^-labeled L81-HP/SBE PR and Gd^3+^-labeled HP-β-CD. Both materials were injected into the IP cavity at 0.03 mmol Gd^3+^/kg. Changes in contrast were evident over 6 h of imaging in the IP space (top), kidney (middle) and liver (bottom). Red arrows indicate the IP space and bladder contrast. Yellow arrows indicate contrast in kidneys. Light blue arrows indicate liver. Images were acquired using a 7T Bruker scanner.

To evaluate the propensity of PR to move from the IP cavity into the blood stream, both gadolinium (Gd^3+^)-carrying L81-HP/SBE PR (Gd^3+^-L81-HP/SBE PR) and Gd^3+^-modified HP-β-CD (Gd^3+^-HP-β-CD) monomer were injected into the IP cavity of normal Balb/c mice and monitored by magnetic resonance imaging (MRI). These materials were covalently modified as stable Gd^3+^:1,4,7,10-tetraazacyclododecane tetraacetic acid (DOTA) chelates. Significant contrast can be seen in the IP cavity of both groups of mice immediately after injection (Fig. 5). Over the course of 6 h, however, there was a progressive reduction of contrast within the IP cavity. Concurrent with this reduction, increased contrast was observed in the kidney and bladder of the HP-β-CD monomer administered mice. In PR-treated mice, there was little contrast evident in the kidney or bladder at any time. In the case of PR treatment, significant contrast was most evident in the liver.

### PR Intraperitoneal Injection Pharmacokinetics

**Figure 6:**
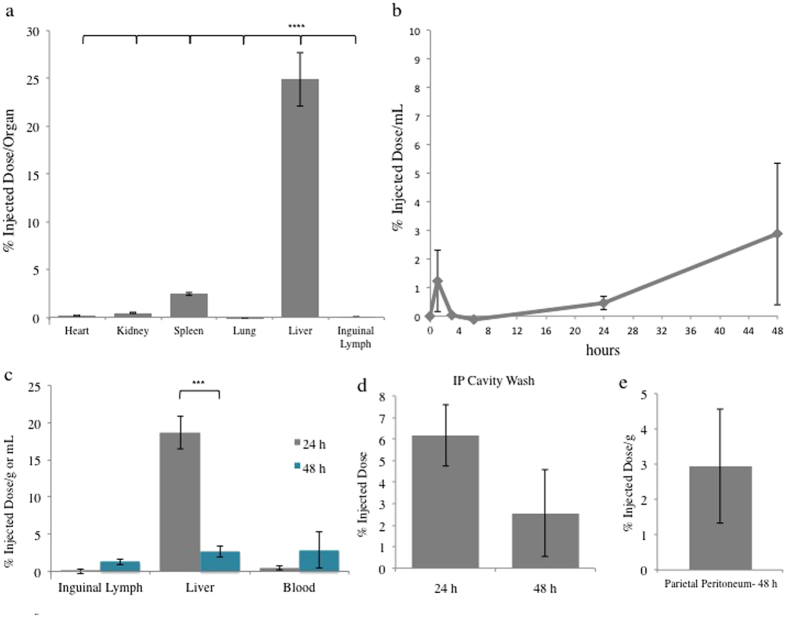
L81-HP/SBE PR pharmacokinetics and biodistribution after intraperitoneal injection. Gd^3+^:DOTA labeled L81-HP/SBE was administered to WT mice by IP injection. Samples were collected at the specified times before HNO_3_ digestion and analysis for Gd^3+^ content by ICPMS. (**a**) PR biodistribution at 24 h revealed predominantly liver deposition (n = 4). (**b**) PR pharmacokinetics showed low blood concentrations over time (n = 4; except at 24 h where n = 7 and 48 h where n = 3). Gd^3+^ concentrations measured in the (**c**) blood, liver and lymph nodes and recovered from the (**d**) IP cavity at 24 and 48 h (n = 4). (**e**) Gd^3+^-containing material that remained adhered to the parietal peritoneum at 48 h (n = 4). ***p = 0.005, ****p = 0.001.

Upon tissue digestion and ICPMS analysis, significant deposition of Gd^3+^-labeled L81-HP/SBE PR material was predominantly found in organs of the reticuloendothelial system (RES) at 24 h. The liver and spleen contained ~25% and ~2.5% of the injected Gd^3+^ dose (ID) per organ, respectively (Fig. 6a). Insignificant levels of material were found in the kidney, lung, and heart (all collecting <1% ID/organ). This is in contrast to the Gd^3+^-HP-β-CD monomer, where the majority of the injected dose was found in the kidneys, with <10% ID/organ depositing in the liver (Supplementary Figure S4). PR concentrations in the inguinal lymph nodes and blood increased between 24 and 48 h, while the concentration in liver dropped significantly, from ~18.6% ID/g to ~2.6% ID/g, over the same timeframe (Fig. 6c). In an effort to identify the fraction of PR dose that had escaped the IP cavity, Gd^3+^-containing material accounting for ~6.2% and ~2.6% of the injected dose was recovered from the IP cavity at 24 h and 48 h, respectively (Fig. 6d). At 48 h, the concentration of PR remaining adhered to the parietal IP membrane, the membrane lining the outer surface of the cavity attached to the abdominal wall, was ~2.9% ID/g (Fig. 6e). Low levels of Gd^3+^ were found circulating in the blood at every time point studied after injection (Fig. 6b). Less than ~4% of the ID is detectable in the blood, on average, between 1 h and 48 h. The average Gd^3+^ concentration rose at time points after 6 h, but at no point over the course of the study were the differences between the blood Gd^3+^ levels statistically significant.

## Discussion

HP-β-CD derivatives have been shown to be therapeutically active in NPC, reducing cholesterol accumulation and extending survival in mouse and cat models of the disease. Neurological disease can be effectively treated using intrathecal administration, however, amelioration of visceral disease is hampered by the poor bioavailability of monomeric HP-β-CD and pharmacokinetics that are largely characterized by rapid renal filtration. We sought to improve the bioavailability of HP-β-CD by utilizing PR drug delivery scaffolds that should hamper renal filtration and enable effective treatment of NPC visceral disease.

PR therapeutic efficacy relative to monomeric HP-β-CD was found to be dependent on the organ of interest. Organs that are known to be unresponsive to monomeric HP-β-CD treatment, such as lung, remain unresponsive to PR administration. Rotaxanation of monomeric HP-β-CD into a PR motif, therefore, did not promote UC clearance from lung and brain tissues, despite an increase in the residence time of the HP-β-CD prodrug in the animals^25^. In the liver and spleen, L81PR treatment diminished the total UC pool to concentrations similar to low dose monomeric HP-β-CD. These findings indicate that PR administrations can be effective at >10-fold lower molar concentrations of agent. In the kidney, this discrepancy increases to >100-fold, showing that IP administration of PR can provide enhanced therapeutic efficacy, even though the PR must cross the IP membrane to enter systemic circulation.

This necessity has been shown to greatly alter the pharmacokinetics and biodistribution of nanomaterials when compared by both IP and IV administration^35^,^36^,^37^. Efflux of nanoparticles across the peritoneum can be slow and lead to an extended biphasic absorption into blood^35^. In addition, IP injection can lead to an enhanced deposition of nanoparticles in the stomach, intestines, and pancreas at the expense of the other visceral organs^35^,^36^. IP injection of micelles has been shown to bias accumulation toward peritoneal membranes and peritoneal tumors relative to IV injection. This also comes at the expense of deposition into the liver^37^. Finally, nanomaterials injected into the peritoneum can precipitate onto the cavity surface of serous membranes and never reach systemic circulation^38^. Alternative routes of administration, including direct access to the blood stream via IV injection, therefore, may be preferred for enhancing PR-mediated cholesterol reduction.

Overall, PR physiochemical characteristics were found to play both decisive and subtle roles in therapeutic efficacy toward UC clearance. Differences in the co-polymer core and/or threading efficiency led to substantial differences in UC reduction efficacy *in vivo*. In both the liver and the spleen, treatment with L81-based materials resulted in significant reduction of the UC burden in NPC^nih^ mice, while treatment with F127PR resulted in negligible therapeutic effect. Further study will be needed to determine the cause of this therapeutic difference, although it does not appear to be an effect of PR molecular weight (Table 1). It is possible that differences in CD threading, with highly threaded L81PR providing more rigid rod-like conformations, are responsible for altering material circulation times and biodistributions such that they are more biased toward liver and spleen deposition. Alternatively, the low threading and increased hydrophilicity of F127-based PR may adversely affect their cellular uptake and promote their elimination by renal filtration. It appears that alteration of surface charge, through the incorporation of charged SBE-β-CD, has its primary effect on PR solubility, since the impact on UC reduction was similar to that of L81-HP PR. Of the two L81-based PR, however, L81-HP/SBE PR performs slightly better, on average, in UC reduction within both the liver and spleen relative to untreated NPC^nih^ mice.

The exact mechanism by which PR mediate cholesterol removal from sites of abnormal sequestration is currently unknown. It is hypothesized that enzymatic degradation within the LE/LY triggers the release of therapeutically active CD monomers from the PR scaffold. PR endcaps are covalently linked to the polymer core by carbamate moieties that have been previously used in the design of prodrugs^39^. There have also been recent reports that PEG-lipid micelles are able to elicit cholesterol efflux, hypothesized to occur through solubilization of existing cholesterol crystals^40^. Given the polymeric nature of PR, it is possible that the mechanism of action is totally or in part governed by its ability to similarly solubilize cholesterol deposits for efflux.

Because of their accumulation within the liver and their ability to reduce cholesterol concentrations in the liver and spleen, L81PR were screened for their effects on CYP isoforms. It is known that nanomaterials can have either inhibitory or stimulatory effects on CYP function properties that have the potential to lead to toxic effects, drug-drug interactions, and recommendations for administration and contraindications^30^. In addition, it has recently been reported that NPC patients have diminished CYP activity^31^. Further exacerbating this deficiency through CYP inhibition could have unanticipated deleterious effects. As patient lifetimes are extended through the effective treatment of neurological disease (e.g., via IT injection of HP-β-CD), visceral disease will also need to be addressed. It is important that these systemically administered agents minimize the impairment of an already dysfunctional CYP system. Thus, the CYP isoforms chosen for this investigation were some of the more prevalent and important species involved in drug metabolism. These included CYPs 3A4, 2C9, and 2D6, isoforms that account for ~30%, ~12%, and ~3% of the CYP content resident in the liver, respectively. Ultimately, these isoforms are responsible for ~50%, ~10%, and ~30%, respectively, of total drug metabolism^41^.

In almost every case, PR inhibit substrate turnover by CYPs in a concentration dependent manner. CYP 2D6 appeared to be the isozyme most adversely affected by incubation with PR scaffolds. High concentrations (10 μM) of both L81-HP/SBE PR and L81-HP PR slowed substrate turnover to 31% and 22% of control, respectively. This was considerably more than the effect on 2C9, where 10 μM PR incubation resulted in turnovers of 55% and 34%, respectively. CYP 3A4 was the least adversely affected by incubation with PR scaffolds, an important finding given its prominence in drug metabolism. Since these findings indicate that PR have the potential to interfere with normal drug turnover, continued evaluation of potential drug-drug interactions *in vivo* are needed in subsequent pre-clinical studies.

These results are clearly different from those obtained through CYP incubation with the PR precursor materials Pluronic L81, HP-β-CD, and SBE-β-CD (Supplementary Figure S7) at similar molar concentrations to those present on PR. CYP 3A4 and 2C9 are largely unaffected by incubation with these materials. Only at the highest concentrations of CD derivatives is there a statistically significant inhibition of substrate turnover, where levels drop to ~80% of control. Again, CYP 2D6 appeared to be most affected by incubation with precursor components. Both 3 μM and 5 μM Pluronic L81, every concentration of HP-β-CD, and higher concentrations (100 μM and 300 μM) of SBE-β-CD produced significant inhibition of substrate turnover by 2D6. These trends do not mirror those seen upon incubation with PR materials, suggesting that PR are acting as intact species and not upon metabolically triggered dethreading.

Finally, these results point to clear differences between the inhibitory potential of the different scaffolds. A main difference between the two scaffolds is the identities of the CD derivatives they carry. In the case of 2C9 and 2D6, PR carrying solely HP-β-CD have a greater impact on substrate turnover than those PR carrying a mixture of HP-β-CD and charged SBE-β-CD. It is possible that the increased negative charge acts to suppress interactions with the Baculosome^®^ plasma membranes or the CYP isoforms themselves. For CYP3A4, however, the opposite trend is true. The interaction of PR with varying CYP isozymes, therefore, is nuanced and dependent on the physicochemical properties of the PR themselves. These insights can be used for the design and synthesis of next generation materials with decreased potential for toxicity.

In order to understand material distribution and bioavailability, we attempted to determine the absorption rate from the IP space of PR and HP-β-CD monomer that were covalently modified with stable Gd^3+^:DOTA chelates. The pharmacokinetics and biodistribution of the dose was monitored thereafter using MRI and periodic ICPMS analysis of Gd^3+^ content. Gd^3+^:DOTA-labeled monomeric HP-β-CD and L81-HP/SBE PR, upon injection into the mouse IP cavity, appear to exhibit loss of IP contrast enhancement at similar rates (Fig. 5). In the case of Gd^3+^:DOTA-labeled monomeric HP-β-CD, this was paired with an increase in contrast in the kidney and bladder. These observations are consistent with migration of the monomeric HP-β-CD dose across the peritoneal membrane and into the bloodstream before renal filtration. In contrast to the monomer, Gd^3+^:DOTA-labeled L81-HP/SBE PR did not appear to any appreciable extent in the kidney or bladder of the mice within the first 6 h of imaging, although enhanced contrast was observed in the liver. This divergence in biodistribution suggests differences in the rates and mechanisms by which the two materials are being transported *in vivo*. These findings are also supported by our Gd^3+^ biodistribution ICPMS data showing PR residence in the liver and monomeric HP-β-CD residence in the kidney 24 h (Fig. 6, Supplementary Figure S8). In addition, the fact that the PR material deposits most significantly within spleen and liver is consistent with the observed cholesterol reduction efficacy in NPC^nih^ mice (Fig. 2).

A direct attempt to study the movement of Gd^3+^:DOTA-modified L81-HP/SBE PR into the bloodstream from the IP cavity was made by studying the Gd^3+^ concentrations in blood over time. Low concentrations of Gd^3+^ were present in the bloodstream over the first 48 h of monitoring, consistent with slow PR transport across the peritoneal membranes. In addition, only ~28% of the injected Gd^3+^ dose could be accounted for in the visceral organs 24 h after PR injection. We infer from these findings that either a significantly impeded movement of the material into the blood stream or lymphatic drainage after recognition by the immune system in the IP cavity is occurring upon IP injection. Rapid excretion of >70% of the injected dose through renal filtration is unlikely due to the minimal amount of PR found in either blood circulation or the kidney, by both MRI contrast and ICPMS.

Low concentrations of PR were found in the local lymph nodes. Significant percentages of injected dose, however, were recovered from the IP space at 24 and 48 h (Fig. 6d). Further, there was significant material deposition on the parietal peritoneum itself that was not recovered by washing procedures (Fig. 6e). This suggests that there is material adherence to the peritoneal membranes, likely throughout the IP cavity, in addition to that which was extracted at 24 and 48 h. The decreasing MRI contrast within the IP cavity despite the presence of significant PR material may be explained by material aggregation and precipitation onto IP membranes as the PBS vehicle is systemically absorbed leading to a PR concentration effect. Regardless, this extended adsorption will result in a bioavailability that is greatly divergent from the HP-β-CD monomer and potentially reducing the overall PR treatment efficacy observed at the single time point evaluated.

In order to treat neurological disease, it will be important to achieve PR access to the brain. Surface modification of PR with materials having a propensity to cross the blood-brain barrier (BBB), or that are capable of engaging BBB-specific receptors, is one potential route toward enhanced uptake. Decoration of PR scaffolds with transferrin or Apolipoprotein E – either via covalent or non-covalent (i.e. avidin-streptavidin) interactions, or by formulation approaches – could facilitate transcytosis across the BBB^42^. Finally, there are polymeric materials, including polysorbate 80, that engender enhanced BBB uptake through osmotic shrinkage mechanisms that could potentially be included in the PR formulations^43^.

The pharmacokinetic profile reported here contrasts with those we have previously described after IV PR administration^26^. L81-HP PR were found to exhibit a biphasic circulation time, with material continuing to circulate at 24 h. By this route of administration, the requirement for peritoneal membrane penetration was eliminated and the entirety of the dose would be bioavailable. Final biodistribution was predominantly found in liver, spleen, and lung (~67%, ~10%, and ~8% ID/organ, respectively) at 24 h. This suggests that IV injection may be preferred for accessing organs, including lung, that were not accessed by IP administration. In addition, PR of varying architectures exhibited greatly enhanced circulation times relative to that of the HP-β-CD monomer. This increased bioavailability of PR via IV administration suggests that this route will be beneficial for cholesterol reduction by achieving a higher circulating concentration of the injected dose.

## Conclusion

Here, we report the first use of PR drug delivery scaffolds to treat NPC *in vivo* using LC/MS/MS to monitor UC accumulation. Our findings show that PR can effectively diminish the total UC pool of visceral organs susceptible to HP-β-CD monomer treatment at greatly reduced molar concentrations when administered IP. Organs that are unresponsive to monomeric HP-β-CD (e.g. lung) are also unaffected by PR administration. In addition to this proof-of-concept, use of PR scaffolds with differing physiochemical properties revealed structure-activity relationships wherein the PR threading extent and polymer core type appear to greatly influence therapeutic efficacy, while the role of negative charge via inclusion of SBE-β-CD derivatives appears to mainly affect PR solubility and not cholesterol efflux activity. In addition, PR scaffolds show a concentration dependent inhibition of three of the most prevalent and important CYP isoforms. These effects are dependent on both the isoform in question and the physicochemical properties of the PR itself. This knowledge will be invaluable in guiding PR candidate selection for clinical translation.

Finally, PR exhibited slow transport across peritoneal membranes and entry into systemic circulation before ultimately depositing largely in the liver and spleen. This is in contrast to the largely renal tropism of monomeric HP-β-CD, showing that rotaxanation greatly alters CD performance *in vivo*. Significant percentages of injected PR doses were found remaining in the PR cavity and adhered to IP membranes, a finding that may suggest a more prolonged effect beyond the 14 d period assessed in this study. Alternative routes of administration that can enhance PR bioavailability may achieve even greater therapeutic effect. Taken together, these data suggest that PR scaffolds hold great promise as potential treatments for visceral disease in NPC patients.

## Methods

### Materials

2-Hydroxypropyl-β-cyclodextrin and ketoconazole were purchased from Sigma Aldrich (St. Louis, MO). 4-Sulfobutylether-β-cyclodextrin was a gift from Ligand Pharmaceuticals (La Jolla, CA). Pluronics L81 and F127 were purchased from BASF (Ludwigshafen, Germany). d_7_-Cholesterol was purchased from Avanti Polar Lipids (Alabaster, AL). Baculosome Plus P450 reagent kits and quinidine were purchased from ThermoFischer Scientific (Grace Island, NY). Isoforms chosen were 3A4, 2C9 and 2D6. All reagents were used as received without further purification or characterization. Sulfaphenazole was obtained from Cayman Chemical (Ann Arbor, MI).

### PR Synthesis and Characterization

PR were synthesized and characterized as previously reported^24^,^25^.

### Animals and Injection Protocol

NPC1^nih^ mice, originally established at the National Institutes of Health, were obtained from Jackson Labs and utilized for the study of therapeutic efficacy^44^. Experiments were carried out at the University of Notre Dame. These mice are characterized by a truncation and premature translation of the NPC1 protein. Mice were bred and genotyped as previously described^45^. Mice of both genders were randomly assigned into groups and treatment was started at 21–23 d of age. Mice received two IP injections of PR or HP-β-CD once per week for two weeks (400 mg/kg or 4000 mg/kg equivalent HP-β-CD concentration) before being sacrificed by CO_2_ asphyxiation at 34–38 days of age. The organs (liver, spleen, kidney, brain, and lung) were harvested and flash frozen. All studies were approved by the IACUC of the University of Notre Dame and performed in accordance with relevant guidelines and regulations.

For pharmacokinetic and biodistribution analysis, male Balb/c mice for MRI imaging were purchased from Charles River (Boston, MA). Injections were done at 5–7 weeks of age with mice weighing 20–24 g. PR materials (10 mg/kg) were administered in sterile PBS by IP injection. Each PR was administered in a 100 μL injection to a group of 4 mice. Blood draws of ~50 μL were taken at 1 h, 3 h, and 6 h after administration. At 24 hours, mice were sacrificed by CO_2_ asphyxiation and exsanguination. Liver, spleen, kidney, lungs, and heart tissues were dissected for *ex vivo* biodistribution analysis. Blood and organs were stored at −80 °C until analysis. At 24 and 48 h, washing and sampling of the IP cavity was performed by injecting 4 mL PBS into the IP cavity before cavity palpitation and removal of the injected fluid^46^. In these mice, the previously stated visceral organs were collected along with the thymus, inguinal lymph nodes and parietal peritoneal membrane. All experiments were performed under an approved protocol from the Purdue Animal Care and Use Committee and performed in accordance with relevant guidelines and regulations.

### Lipid Extraction, Saponification, and Derivitization

Organ samples were weighed and transferred into Precellys homogenization vials (Cayman Chemical, Ann Arbor, MI). The entire organ was used in every case, except for liver samples where ~150–200 mg were used, and when examining kidneys where a single kidney was utilized. Samples were then homogenized (6500 g, 3 × 90 s). This process was repeated after centrifugation for 2 min at 1500 × g. Samples were again centrifuged before addition of 0.5 mL 0.5 M KOH for saponification. After vortexing, samples were heated at 45 °C for 2 h with shaking. Next, samples were extracted with 2 portions of 1 mL hexane. This extract was diluted 1:4 and 50 μL of each sample was transferred into glass vials. To this 50 μL aliquot was added 10 μL of d_7_-cholesterol (40 ng/μL) as internal standard for quantification. Samples were dried completely under a stream of N_2_ (g).

For analysis, samples were derivitized to picolinic ester derivatives as described by Honda *et al*.^32^ Briefly, a stock solution was generated consisting of 2-methyl-6-nitrobenzoic anhydride (0.1 g), 4-dimethylaminopyridine (30 mg), picolinic acid (80 mg) and THF (1.5 mL). An aliquot of derivatization stock solution (150 μL) was then added to each dried cholesterol extract and vortexed before addition of 20 μL triethylamine. Samples were incubated at 20 °C on a shaking plate for 30 min before being centrifuged (1500 × g, 3 min) and transferred to LC injection vials.

### LC/MS/MS analysis

LC/MS/MS analyses were performed using an Agilent Triple Quadrupole LC/MS/MS (Santa Clara, CA). Mobile phases (flow rate: 0.8 mL/min) were A: H_2_O + 0.1% formic acid (FA) and B: 50:50 ACN:MeOH + 0.1% FA, with a gradient sequence given in Table 3. A Vydac C4 column (Grace, Columbia, MD) was used as stationary phase. The charging voltage was 1000 V and the capillary voltage was 4000 V. The nebulizer gas temperature was 325 °C, the gas flow was 8 L/min, and the nebulizer pressure was 45 psi. The sheath gas heat and flow rate was 250 °C and 7 L/min, respectively. Injection volumes were 0.5 μL and the total run time was 15 min. Transitions monitored were *m/z* 492-386 for cholesterol and *m/z* 499-393 for d_7_-cholesterol. Ion polarity was positive. Three sequential injections of wash solution (ACN or MeOH) were done after every six experimental samples. Liver samples were extracted, derivatized, and analyzed in two separate experiments and found to differ by ≤5.8%. All subsequent cholesterol analyses were done once.

### Cytochrome P450 Isozyme Turnover

These studies were done by characterizing the cleavage of known CYP substrates in the presence of PR compounds and controls. The CYPs exist as single isoforms in commercially available microsomes isolated from transfected insect cells known as Baculosomes. Baculosomes (isoforms 3A4, 2D6, and 2C9) were utilized to study fluorogenic substrate turnover of specific isoforms as per manufacturer instructions. Fluorogenic substrates utilized were 7-ethoxymethoxy-3-cyanocoumarin and 7-benzyloxy-methyloxy-3-cyanocoumarin. Briefly, baculosomes and NADP^+^ were mixed and diluted to a final concentration of 10–20 nM depending on the isoform. Stock solutions of inhibitor, test sample (PR or starting material), or solvent control were made and serially diluted to 2.5x the desired concentration. Next, 50 μL of Baculosome stock and 40 μL of test sample stock were mixed in a 96-well plate and incubated for 10 min. Finally, 10 μL of a stock solution containing fluorogenic turnover substrate and NADP^+^ was added to each well to initiate the reaction. Final concentrations were 10 nM (2C9 and 2D6) or 5 nM (3A4) for baculosomes, 3 μM–10 μM for PR, 30 μM for sulfaphenazole, 10 μM for ketoconazole, 10 μM for quinidine, 10 μM for substrate and 300 μM for NADP^+^. Substrate turnover was monitored every 60 s for 1 h using a Biotek Neo plate reader (Winooski, VT). The excitation and emission wavelength were set at 415 nm and 460 nm, respectively. All samples were run with 3–6 biological replicates and reported as a percent of the uninhibited fluorogenic substrate control.

### Magnetic Resonance Imaging

Male Balb/c mice for MRI imaging were purchased from Charles River (Boston, MA). Injections were done at 5–6 weeks of age with mice weighing 20–22 g. Isoflurane gas was used for anesthesia. Body temperature and respiration were monitored for the duration of the experiment using a Small Animal Monitoring and Gating System (SA instruments, Stony Brook, NY). Mice were anesthetized and imaged before injection and for 1 h immediately following injection. The administered Gd^3+^ concentration was 0.03 mmol/kg. Additionally, mice were imaged at 3 h and 6 h post-injection. Finally, mice were sacrificed by CO_2_ asphyxiation and exsanguination at 24 h after PR injection. Liver, spleen, kidney, lungs, and heart tissues were dissected for *ex vivo* analysis of PR biodistribution. All experiments were performed under an approved protocol from the Purdue Animal Care and Use Committee.

MRI images were obtained using a Bruker 7 T small animal scanner using a T1-FLASH pulse sequence. Echo time was 1.532 ms, repetition time was 500 ms and NEX was 2 with single repetitions. Twenty-five slices were used with a flip angle of 30.0° and a slice thickness of 1.00 mm.

### Tissue Digestion

Organ samples were thawed, weighed, and homogenized before digestion with 100–200 μL 70% HNO_3_ at 70 °C for 24–48 h. Livers and kidneys required longer digestion times than did lungs, spleens, and hearts. Harvested IP fluid was lyophilized and re-solubilized with H_2_O (200 μL) to concentrate the sample. A 20 μL aliquot of this solution was added to 100 μL of 70% HNO_3_. After digestion, hearts were diluted to 5 mL total volume. Spleen, kidney, and lung samples were diluted to 5 mL and then further by 1:10. Livers were diluted to 10 mL volumes and further by 1:15. Digestion of blood samples was achieved (5–20 μL) by addition of 70% HNO_3_ (100 μL). Samples were incubated at 70 °C for 16 h–24 h to give pale yellow solutions before dilution to 5 mL. Final solutions for ICPMS analysis were filtered through 0.2 μm PTFE syringe filters (Macherey-Nagel, Bethlehem, PA). Final HNO_3_ concentrations of all samples was 2%.

### Inductively Coupled Mass Spectrometry Analysis

The ^156^Gd and ^158^Gd inductively coupled argon plasma mass spectrometry (ICPMS) results were obtained using an ELEMENT-2 (ThermoFinnigan, Bremen, Germany) mass spectrometer, with a Netbuie data system upgrade, in the medium resolution mode (which minimizes interferences). R = M/ΔM ~3500. The mass offset program was used with the ^40^Ar ^40^Ar peak at m/z 79.7242 as the locked mass in order to prevent magnet shifts with time. The Ar sweep gas and N_2_ of the Aridus was adjusted for maximum peak height and stability using ^7^Li, ^115^In and ^238^U peaks obtained from a Merck multi-element standard (1 ng/ml, Merck & Co.). Typical sweep gas values were 16–20 L/min for N_2_ and 2.5–3.5 L/min for Ar. The tuning peaks were enhanced by adjusting the lenses prior to instrument calibration.

A 10 ppb Gd standard was used (Exaxol, Clearwater, FL) to determine the mass offsets and the ^40^Ar ^40^Ar signal was set as the locked mass peak. An Aridus desolvating system with a T1H nebulizer (Teledyne Cetac, Omaha, NE) was used to introduce the samples into the plasma to enhance sensitivity and reduce oxide and hydride interferences. The sample uptake rate was approximately 60 μL/min with an Ar nebulizer gas flow rate of 1 L/min. The spray chamber was heated to 80 °C to help reduce the formation of solvent droplets. An ASX-100 autosampler (Teledyne Cetac, Omaha, NE) was used. Each sample was scanned with 3 runs and 30 passes with a wash time of 2 minutes and a take up time of 1 minute.

### Statistics

Cholesterol reduction statistics were generated using Graphpad Prism (La Jolla, CA). Efficacy and biodistribution data was analyzed using ANOVA. Efficacy was evaluated in comparison to untreated NPC controls. Concentration changes from 24 h to 48 h were analyzed using a Students T-test. All values are presented as mean ± s.e.m. *p < 0.05, **p < 0.01, ***p < 0.005, ****p < 0.001.

## Additional Information

**How to cite this article:** Collins, C. J. *et al*. Pluronic based β-cyclodextrin polyrotaxanes for treatment of Niemann-Pick Type C disease. *Sci. Rep.* **7**, 46737; doi: 10.1038/srep46737 (2017).

**Publisher's note:** Springer Nature remains neutral with regard to jurisdictional claims in published maps and institutional affiliations.

## Supplementary Material

Supplementary Information

## Figures and Tables

**Figure 1:**
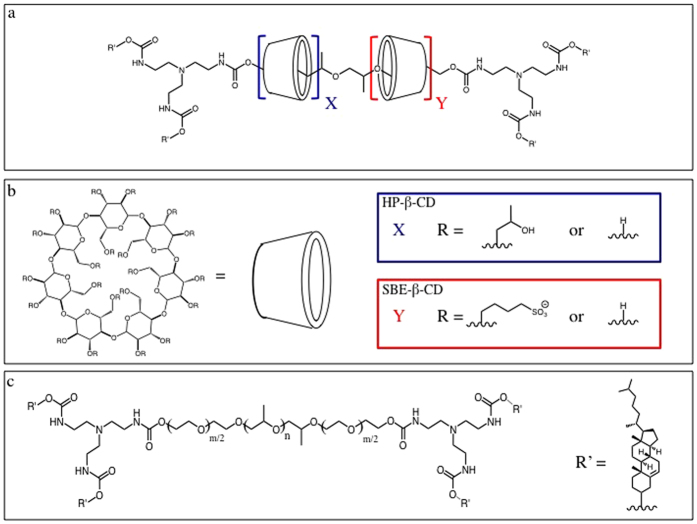
Molecular Structures of PR Components. The overall PR molecular structure (**a**) consists of cyclodextrin-derivative macrocycles non-covalently threaded onto tri-block copolymer cores. CD macrocycles (**b**) will consist of either HP-β-CD (X) alone or a mixture of HP-β-CD and SBE-β-CD (Y). Tri-block copolymer cores (**c**) consist of a central PPG block and outer PEG blocks of varying lengths. Terminal cholesterol substituents serve to block the CD macrocycles from dethreading from the polymer core. L81: m = 6.25, n = 43; F127: m = 200, n = 65.

**Table 1:** Physiochemical properties of PR family members.

PR	PR MW (g/mol)	% CD Threading	Total # CD	# HP-β-CD (X)	# SBE-β-CD (Y)	Core MW (g/mol)	Core PEG (m)	Core PPG (n)
L81 HP	24 kDa	61%	13	13	—	2.8 kDa	6.25	43
L81 HP/SBE	47 kDa	102%	22	7	15	2.8 kDa	6.25	43
F127 HP	31 kDa	34%	11	11	—	12.6 kDa	200	65
F127 HP/SBE	32 kDa	31%	10	6	4	12.6 kDa	200	65

X, Y, m, and n refer to the subscripts appearing in Fig. 1.

**Table 2:** LC/MS/MS cholesterol analysis inter-assay precision.

	WT Control	HP-β-CD (4000 mg/kg)	HP-β-CD (400 mg/kg)	L81 HP	L81 HP/SBE	F127 HP	F127 HP/SBE	NPC Control
Analysis 1 mean ± SD (mg/organ)	1.40 ± 0.16	2.51 ± 0.31	9.0 ± 1.8	14.1 ± 3.1	11.6 ± 1.4	8.9 ± 1.4	8.5 ± 2.1	12.2 ± 1.6
Analysis 2 mean ± SD (mg/organ)	1.36 ± 0.19	2.49 ± 0.31	9.0 ± 1.6	14.4 ± 3.1	11.5 ± 1.9	8.9 ± 1.6	8.8 ± 2.2	11.5 ± 1.9
Percent Difference	3.12	0.69	0.84	1.82	0.82	0.48	2.93	5.85

**Table 3:** LC/MS/MS Solvent Timetable.

Time (min)	Solvent A (%)	Solvent B (%)
0	50	50
1	50	50
5	0	100
10	0	100
11	50	50
15	50	50

Solvent A: H_2_O + 0.1% FA and Solvent B: 50:50 ACN:MeOH + 0.1% FA.
