# Supplementary material for: Pluronic based β-cyclodextrin polyrotaxanes for treatment of Niemann-Pick Type C disease
Source: Sci Rep. 2017 Apr 28;7:46737. doi: 10.1038/srep46737 (PMC5408228; doi:10.1038/srep46737)
Supplement: Supplementary Information [file srep46737-s1.docx]

Supplementary Information

Pluronic based β-Cyclodextrin polyrotaxane formulation for treatment of Niemann-Pick Type C disease

Christopher J. Collins†, Bradley P. Loren†, Md Suhail Alam§,∥, Yawo Mondjinou†, Joseph L. Skulsky†, Cheyenne R. Chaplain†, Kasturi Haldar§,∥, David H. Thompson†,‡,⊥*

†Department of Chemistry, Purdue University, Multi-disciplinary Cancer Research Facility, 1203 W. State Street, West Lafayette, Indiana 47907, United States

‡Purdue University Center for Cancer Research, 201 S. University Street, West Lafayette, Indiana 47907, United States

§Boler-Parseghian Center for Rare and Neglected Diseases, University of Notre Dame, Notre Dame, IN 46556, USA.

∥Department of Biological Sciences, University of Notre Dame, 103 Galvin Life Sciences, Notre Dame, IN 46556, USA.

⊥Weldon School of Biomedical Engineering, Purdue University, 206 S. Martin Jischke Drive, West Lafayette, Indiana 47907, United States


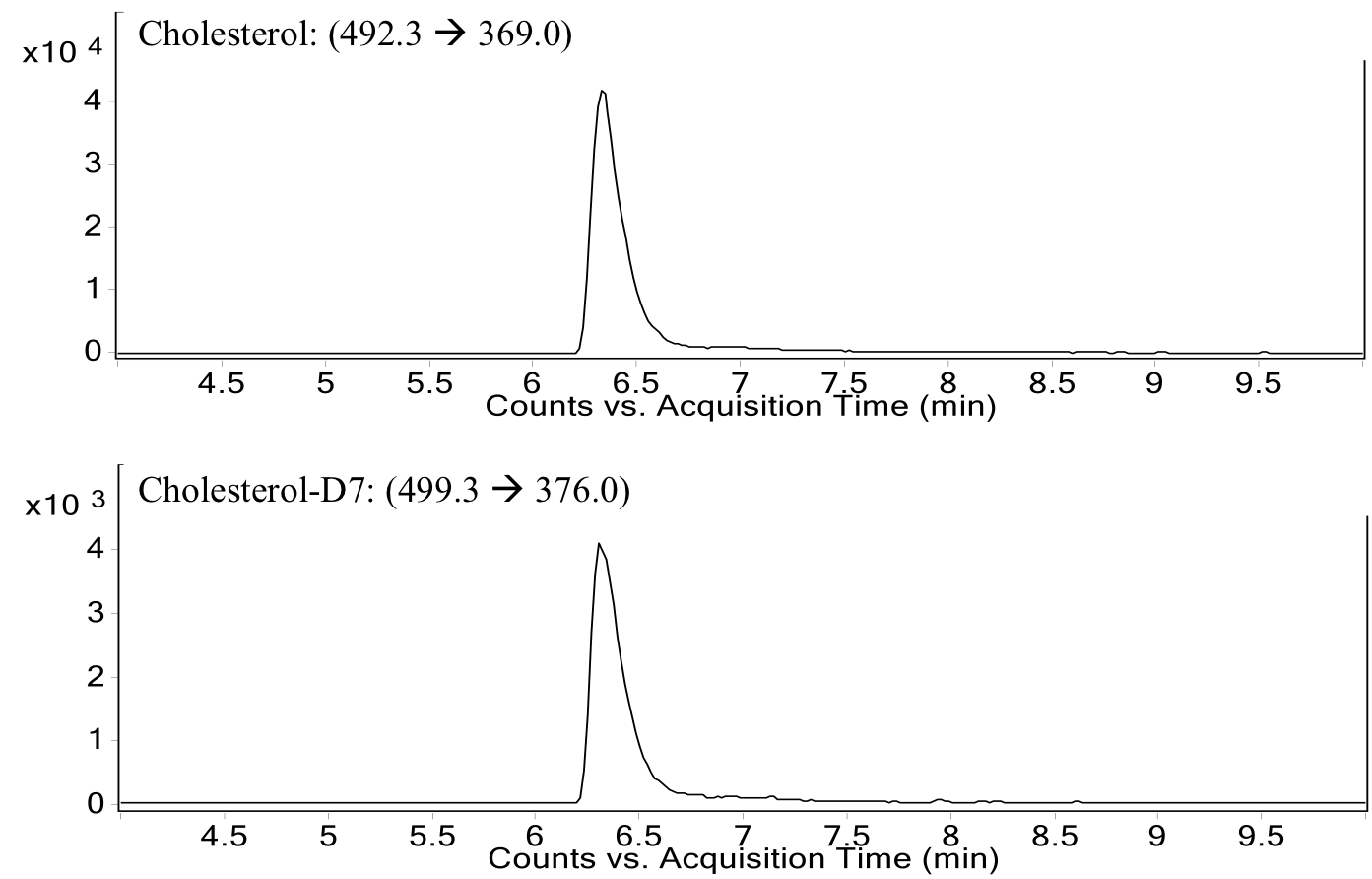


**Supplementary Figure S1: Representative Chromatogram of Cholesterol and Cholesterol-D7.** LC/MS/MS analysis was utilized to quantify cholesterol concentrations in animal tissues. Transitions for cholesterol (top) and isotopic internal standard cholesterol-D7 (bottom) are shown at the top of each chromatogram.


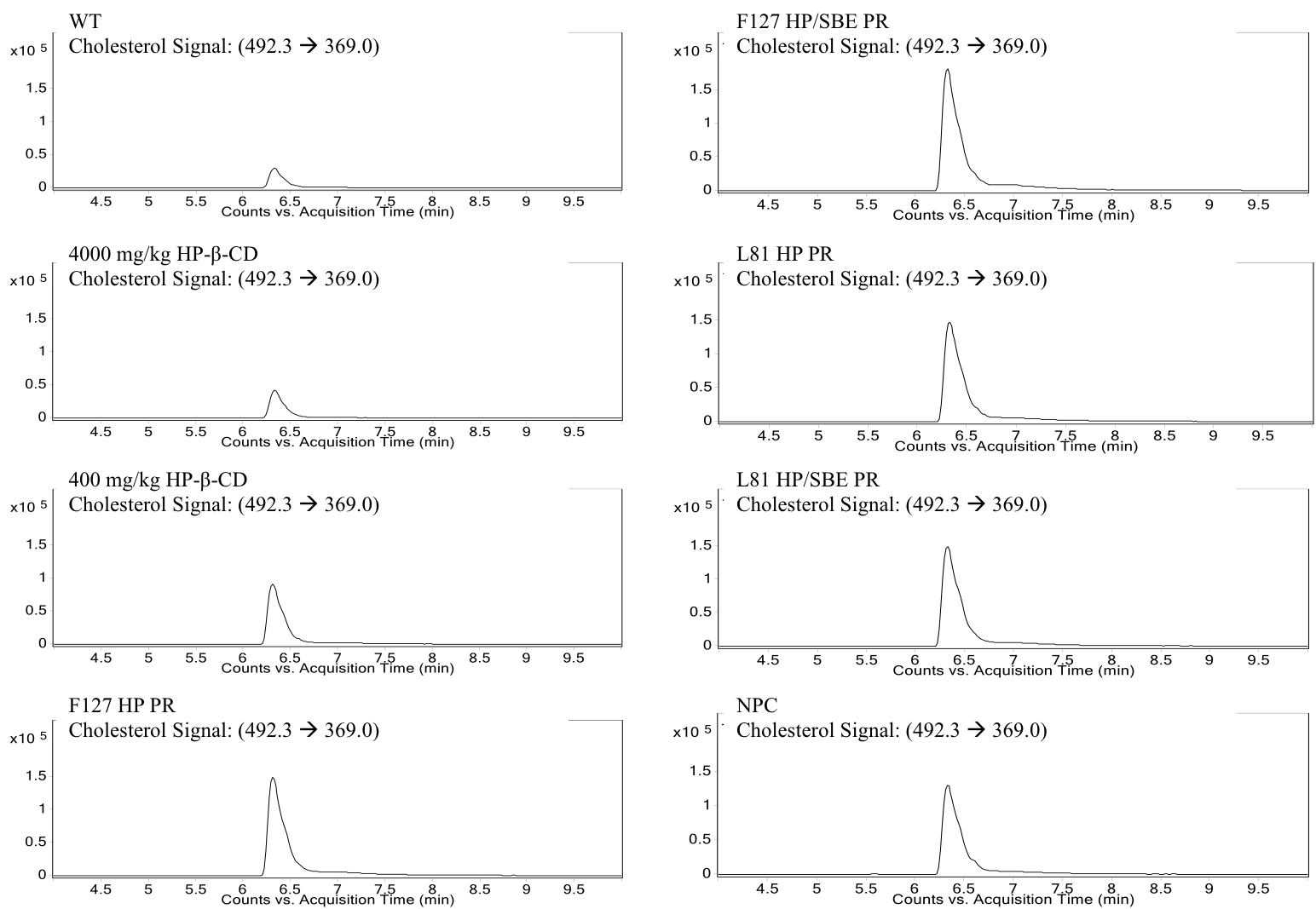


**Supplementary Figure S2: Representative Cholesterol Chromatograms Derived from Various Treatment Groups.** LC/MS/MS chromatograms for each sample are shown. Sample and mass transition information are listed in the top left of each chromatogram.


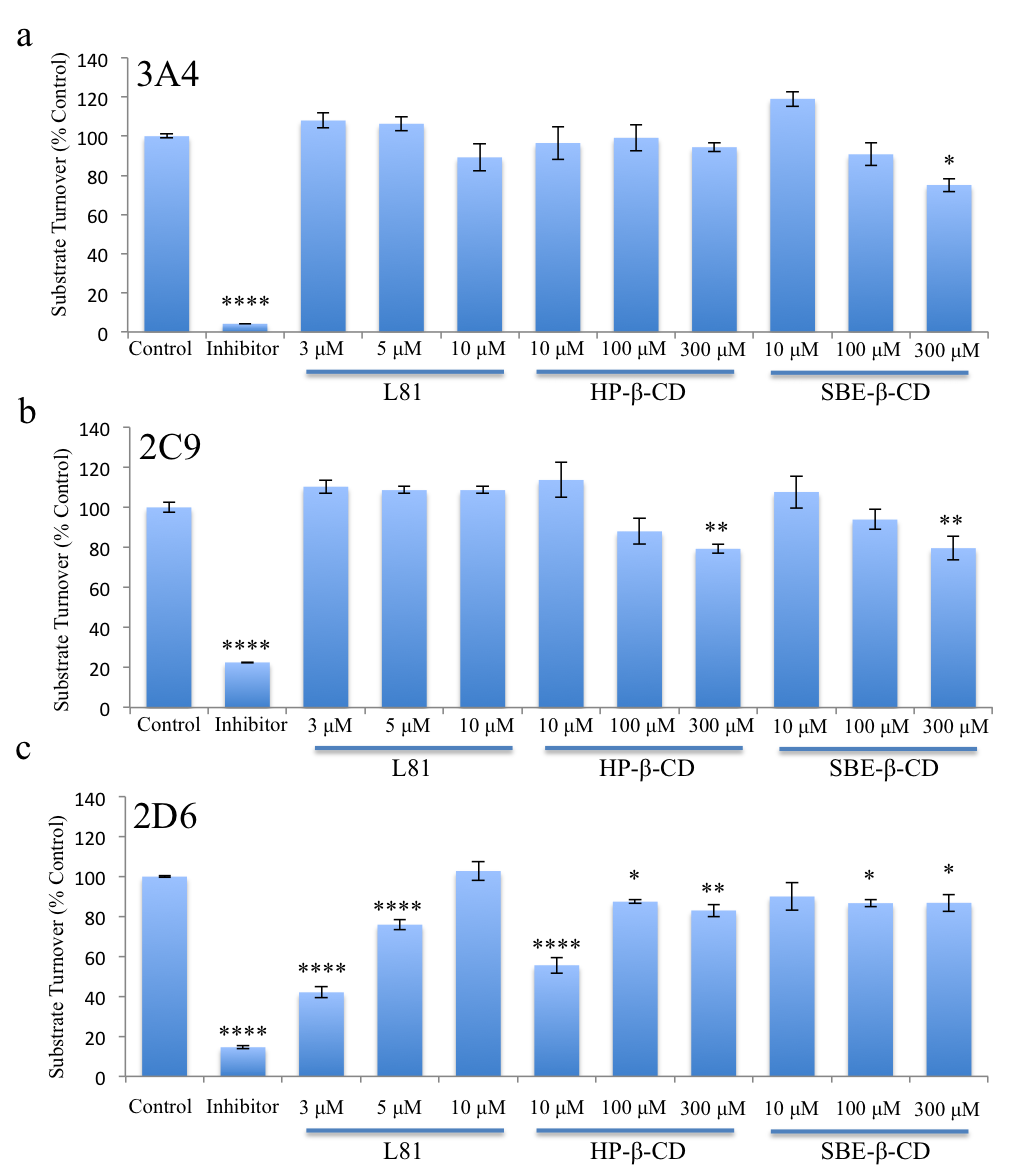


**Supplementary Figure S3: Cytochrome P450 Isoform Turnover: Controls** Isoforms studied included (a) 3A4, (b) 2C9, and (c) 2D6. Studies characterized the cleavage of fluorogenic CYP substrates in the presence of PR starting materials or known inhibitors. The intensity of fluorescence indicates the extent of CYP activity perturbation. Experiments were done in triplicate and expressed as a percent of uninhibited control. *p = 0.05, **p = 0.01, ***p=0.005, **** p = 0.001.


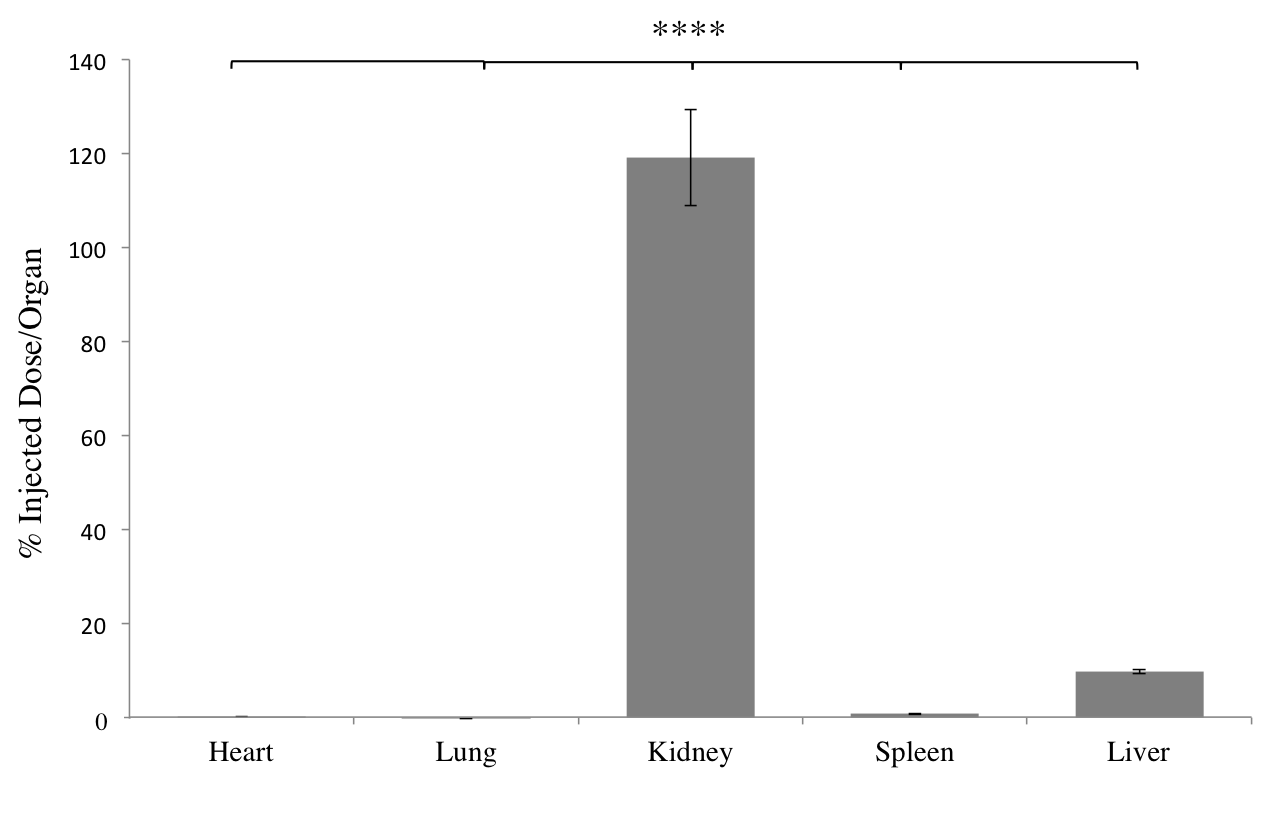


**Supplementary Figure S4: Biodistribution of HP-Gd^3+^ 24 h After Intraperitoneal Administration.** Gd^3+^:DOTA labeled HP-B-CD was administered to WT mice by IP injection. Samples were collected at 24 h before HNO_3_ digestion and analysis for Gd^3+^ content by ICPMS. Biodistribution at 24 h is revealed to consist predominantly of kidney deposition (n = 3). **** p = 0.001.


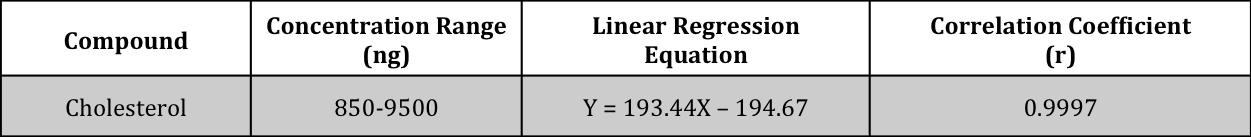


**Supplementary Table S1: LC/MS/MS cholesterol analysis assay linearity.** Increasing concentrations of cholesterol were analyzed by LC/MS/MS to determine linearity over the specified concentration range.
